# Preliminary evidence that remembering together can help younger adults recall the positive

**DOI:** 10.3389/fcogn.2026.1814611

**Published:** 2026-06-22

**Authors:** Marie C. Diagne, Leonard Faul, Suparna Rajaram, Elizabeth A. Kensinger

**Affiliations:** 1 Department of Psychology and Neuroscience, Boston College, Chestnut Hill, MA, United States; 2 Academic Research Services, Boston College, Chestnut Hill, MA, United States; 3 Department of Psychology, Stony Brook University, Stony Brook, NY, United States

**Keywords:** aging, collaborative memory, emotional memory, valence bias, young adults

## Abstract

Emotional information is often more likely to be recalled than neutral information. Among emotional information, there can also be valence biases in memory, with negative content remembered better than positive content or vice-versa. With regard to whether collaborating with others enhances negative memory biases or positive ones, findings in young adults are mixed. Moreover, little is known about how collaboration affects valence biases in older adults' memories, even though it is well documented that older adults often show positive memory biases on individual recall tasks. The present study examined the effect of collaborative retrieval on young and older adults' valence memory biases, by assigning young and older adults to individual-recall or collaborative-recall conditions prior to completing an individual recall task. In the collaborative recall condition, groups were composed of same age-group partners, all young adults or all older adults. Importantly, the recall tasks gave participants three category labels to recall at once, to cue memories in a manner that encouraged the intermixing of differently valenced content. Collaborative retrieval enhanced young adults' memories for all valences, but enhanced memory for positive valence to the greatest extent, resulting in a strong positive memory bias after collaboration. Older adults showed a positive memory bias regardless of whether or not they collaborated. These results emphasize that the positive memory biases typically seen in older adults can be elicited in young adults, if they collaborate with one another.

## 1 Introduction

Collaborative retrieval refers to the process of remembering together in groups. Collaborative retrieval is common after experiencing emotional events. Often, we seek to discuss these events with friends, co-workers, or family. Despite being a commonplace occurrence in everyday life, most of the research on emotional memory has considered it as an individual cognitive process [see recent review of emotional memory by Faul et al. (2024)]. The present study contributes to a nascent literature examining how collaborative retrieval affects emotional memory.

Over the past decade, a few studies have examined how collaboration affects younger adults' emotional memory retrieval. These studies have revealed that emotional memory enhancements can persist after collaborative retrieval (Choi et al., 2017; Jin et al., 2025; Maswood et al., 2019; Kensinger et al., 2016). However, the results have been mixed in terms of the valence of content remembered best after collaboration. For instance, Choi et al. (2017) reported that collaborative retrieval enhanced younger adults' negativity bias in memory, whereas Maswood et al. (2019) found that collaboration led to more positive memories. These studies differed in a number of ways. For example, a negative bias was observed for categorized content that was non-autobiographical in nature (Choi et al., 2017) whereas a positive bias was observed for autobiographical memories (Maswood et al., 2019; Rajaram, 2024a). We reasoned that this distinction may arise from an important difference that is related to whether positive and negative content was being simultaneously cued. In the design of Choi et al. (2013), 2017), participants studied categorized sets of stimuli, with items within each set sharing the same valence (e.g., the “pets” category contained all positive items, the “tools” category contained all neutral items, and the “funeral” category contained all negative items). Although participants could recall items in any order, people's tendency is often to cluster the recall of information (e.g., Gates, 1917; Tulving, 1974; Greeley and Rajaram, 2023), and because category sets were all of one valence, clustering by either category or valence would lead to blocked retrieval of valenced information. Furthermore, given the evidence that young adults are prone to discussing negative events with one another (e.g., Baumeister et al., 2001; Rimé et al., 1992; Rimé, 2007), together these processes likely give rise to a negativity bias in individual recall (Choi et al., 2013; reviewed by Williams et al., 2022) that is enhanced in collaborative recall (Choi et al., 2017; see also Maswood et al., 2019 and Rajaram, 2024a for related discussion).

For autobiographical content (as in Maswood et al., 2019), the content by nature remains intermixed with reference to valence during recall because autobiographical recall is likely to be organized around the structure of the story or the experience, not just around the semantic content or valence of details. Hence, different valences may be intermixed to a greater extent in autobiographical recall than when using nonautobiographical content such as categorized lists. In other words, collaboration may result in positivity biases when the discussion encourages retrieval of positive, negative and neutral content in a more intermixed fashion. In the present study, we adapted the design of Choi et al. (2017) where we encouraged intermixed recall of content from differently-valenced categories. We sought to determine whether, with that design, collaboration among younger adults would lead to a negativity bias in memory (as in Choi et al., 2017), a positivity bias in memory (as in Maswood et al., 2019), or neither.

A second goal of the present study was to explore how collaborative retrieval would affect older adults' emotional memories. Emotion is one factor known to benefit memory not only in younger adults but also in older adults (Kensinger, 2009). When tested in non-collaborative memory paradigms, older adults show an emotional enhancement of memory (EEM) over delays long enough to include time for memory consolidation (e.g., Ford et al., 2021) and also over short delays (e.g., Garcia et al., 2023). Although the EEM is preserved with aging, there can be age-related shifts in the valence of emotional content that is remembered best, with older adults being more likely than younger adults to remember positive content better than negative content (reviewed by Mather and Carstensen, 2005; see meta-analysis by Reed et al., 2014; Carstensen and DeLiema, 2017). Research on collaborative memory in aging is still a relatively understudied topic relative to a large literature that has focused on age-related changes in memories remembered individually (see Henkel and Kris, 2017 for review). To our knowledge, only one study (Barber et al., 2017) has directly compared the effects of collaborative retrieval on young and older adults' memories for negative, positive, and neutral content. This study revealed that while some effects of collaboration were similar in young and older adults (e.g., collaborative inhibition, which refers to the reduction in the overall group-level of recall for collaborating groups compared to control groups), there was a positivity bias in older adults' memories regardless of collaborative condition. Younger adults recalled significantly more of the negative or neutral pictures than the older adults, but there were no significant differences between the age groups for their recall of the positive pictures. The present study explored whether older adults' positivity bias is maintained, exaggerated, or dissipated by collaboration when the design encourages the mixing of valenced content during collaborative discussion.

## 2 Methods

### 2.1 Participants

We enrolled 101 participants: 62 young adults (ages 18–35) and 39 older adults (ages 60+) from across the United States for this online study. Recruitment was done via social media advertisements, fliers posted around the greater Boston area, emails, and word-of-mouth from former participants. We excluded 10 participants in our analyses, four younger adults (YA) and one older adult due to incomplete data, three YA due to technical issues during the collaboration zoom meeting or experimenter error, and two who were a duplicate enrollment (see Table 1 for demographics of the final sample). With our final sample size of 53 young adults and 38 older adults, we achieved 80% power to detect a medium-to-large (0.39) effect size for an interaction between the between-group variables (age, collaboration) and the within-group variable (valence), as determined in G^*^Power (Faul et al., 2007) using the recommended (Cohen, 2013) estimates for effect sizes.

**Table 1:** Participant demographics.

Age group	Collaborative condition	Age (mean, SD)	Years of education (mean, SD)	Gender (Number of female, male, non-binary)
Young	Individual (*N* = 24)	22.7, 4.59	14.42, 1.95	21F, 3M
	Collaborative (*N* = 29)	21.5, 4.25	14.9, 2.6	22F, 7M
Older	Individual (*N =* 17)	69.4, 5.27	18.35, 2.03	9F, 8M
	Collaborative (*N* = 21)	72.5, 5.44	17.05, 2.31	16F, 5M

Older adults had significantly more education than younger adults (*t*[89]=6.50, *p* < 0.001), likely because many younger adults were still enrolled in undergraduate or graduate programs. Within age groups, individual and collaborative groups did not differ significantly in age or years of education or in gender distribution (all *p* > 0.05).

All participants were prescreened to exclude those with a self-reported history of psychiatric or neurological disorders or for diagnosis of clinical depression or an anxiety disorder. Participants were assigned to either the individual condition, or the collaborative condition where they were scheduled in groups of similar-aged participants (i.e., younger adults collaborated with other younger adults; older adults collaborated with other older adults). If only three or two participants arrived for a collaborative session, we still allowed the group to proceed with that group size. Thus, collaborative groups consisted of two (two YA groups), three (four YA groups and five OA groups) or four (five YA groups or three OA groups) participants (six older adults and seven young adults did not return for the individual test the next day, which is why these numbers differ from those in Table 1). Because dyadic recall may be different than larger-group recall (Basden et al., 2000; Thorley and Dewhurst, 2006), all analyses were repeated without the two-participant groups included; this did not change any patterns of results. All participants were compensated with an electronic payment of $23 ($15/h) upon completion. All procedures were approved by the Boston College Institutional Review Board.

### 2.2 Materials

#### 2.2.1 Stimuli

The stimuli were from Choi et al. (2013, 2017) and comprised 45 categorized lists (15 negative categories such as predators; 15 positive categories such as pets; 15 neutral categories such as plants), each consisting of 8 exemplars that included a photo object and its verbal label (e.g., kitten) (Choi et al., 2013). Examples of neutral categories include: actions, farm animals, footwear, geography, transportation, weather; examples of positive categories are: baby, jewelry, flowers, pets, currency; and negative category examples are: bathroom items, body organs, dental instruments, dirty things, spiders.

The categories and exemplars had previously been normed by younger adults (see Choi et al., 2017). However, because older adults had not previously normed the stimuli, in parallel to conducting the main experiment, we collected norming data from 79 participants (40 young adults, 39 older adults) who rated all images on valence, arousal and self-relevance. These normative data were acquired simultaneously (though in different participants) with conduct of the memory experiment and were used to determine which categories would be included in final analyses of the memory experiment.

#### 2.2.2 Normative ratings of stimuli

Normative data are available on OSF: https://osf.io/59nwr(Norming_data_OSF.csv). Valence ratings were averaged for all items of a particular category, and those valence ratings were compared for the positive, negative, and neutral categories. Valence ratings differed significantly by category condition, as expected (*F*[2,42] = 73.80, *p* < 0.001), with Negative category items rated lower than Neutral (*t*[14]=19.89, *p* < 0.001) and Positive category items (*t*[14]=27.43, *p* < 0.001). Valence ratings were more positive for Positive category items than Neutral category items (*t*[14]=15.11, *p* < 0.001).

Arousal ratings were also averaged for all items within each category and compared across positive, negative, and neutral category conditions. As intended, negative and positive items did not differ significantly in arousal (*t*[14] = 0.20, *p* = 0.847), and both were rated as more arousing than neutral items (negative vs. neutral: (*t*[14] = 2.75, *p* = 0.016; positive vs. neutral: (*t*[14] = −4.65, *p* < 0.001))). This suggests that emotional content (whether positive or negative) elicits higher arousal than neutral content, but the two emotional valences are equally potent in their intensity.

Self-relevance ratings revealed that negative items were rated as significantly less relevant than both positive (*t*[14] = −3.47, *p* = 0.004) and neutral items (*t*[14] = −2.19, *p* = 0.046). Neutral and positive items did not differ in perceived self-relevance (*t*[14] = −0.19, *p* = 0.854).

#### 2.2.3 Category selection

Although these normative data were generally aligned with the expected patterns, upon looking at all of the categories, we noticed that some categories were rated differently between the young and the older adults, or were outliers for their intended valence category. For example, participants rated “geography” more positively than expected, and there were age differences in how negatively individuals rated “in a school.” We therefore reduced the full set of 15 categories per valence to the best 12 categories per valence, and only focused analyses on those 36 categories. We excluded three categories from each valence group (Positive: in a restaurant, jewelry, fish; Negative: spiders, bathroom, body organs; Neutral: geography, weather, in a school).

For the final set of 36 categories, negative category exemplars were rated significantly lower in valence than both neutral (*t*[11] = 35.54, *p* < 0.001) and positive exemplars (*t*[11] = 46.20, *p* < 0.001), with neutral exemplars also rated significantly lower in valence than positive exemplars (*t*[11] = 19.68, *p* < 0.001). For arousal, negative and positive exemplars did not differ significantly from one another (*t*[11] = 0.65, *p* = 0.527), and both elicited higher arousal than neutral exemplars negative vs neutral: (*t*[11] = 3.60, *p* = 0.004); positive vs neutral: (*t*[11] = 4.67, *p* < 0.001). Self-relevance ratings showed negative exemplars as significantly less relevant than both positive (*t*[11] = 4.11, *p* = 0.002) and neutral exemplars (*t*[11] = 2.36, *p* = 0.038), with no significant difference between neutral and positive exemplars (*t*[11] = 1.23, *p* = 0.244). There were no significant age differences in valence, arousal, or self-relevance ratings (all *p* > 0.15).

This manuscript reports the memory results for only the best 36 categories (12 of each valence) in the main body of the paper. Results for all 45 categories (15 of each valence) are provided in Supplementary materials.

### 2.3 Procedure

**Figure 1:**
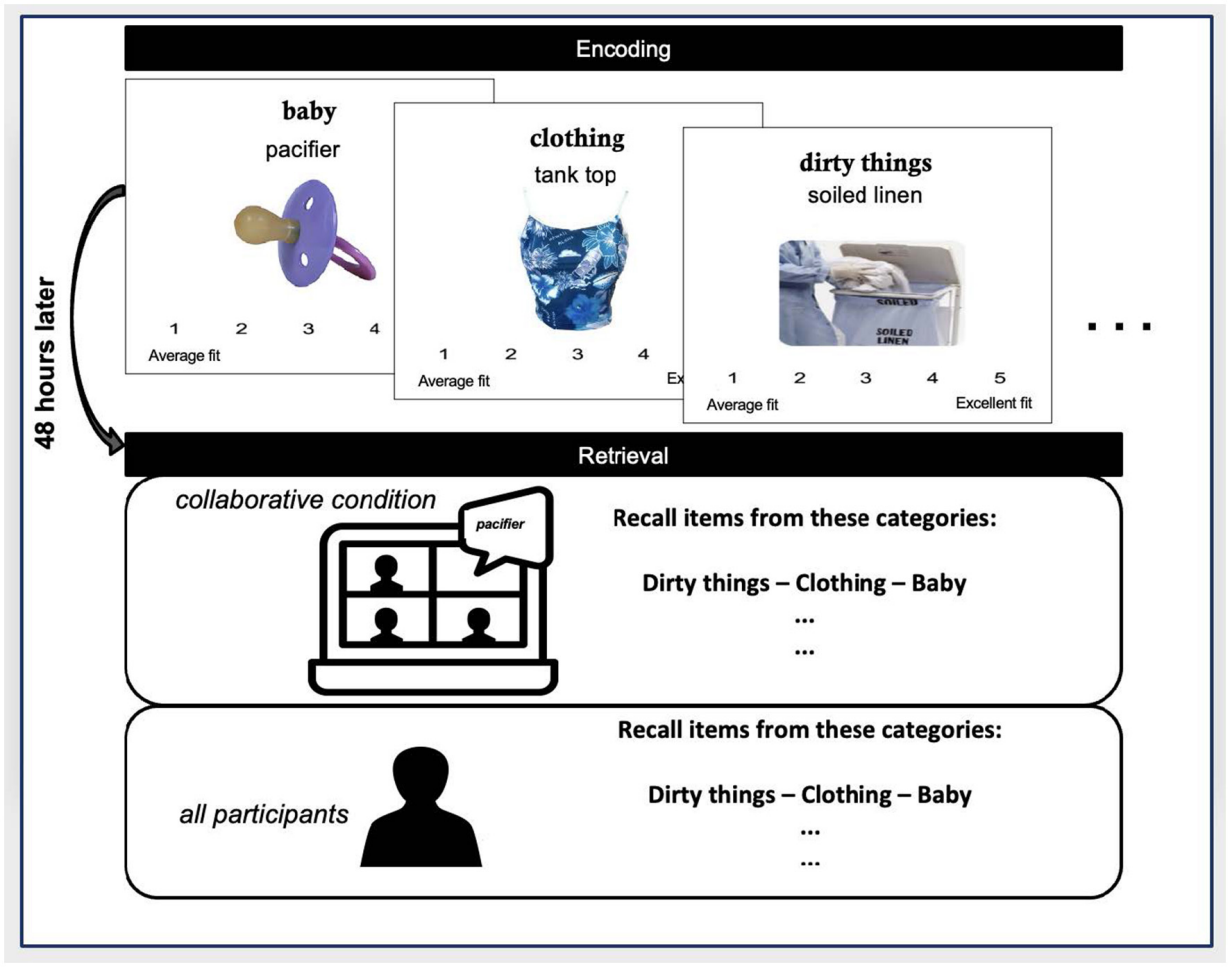
Task procedure. At encoding, participants viewed category labels and exemplars that were positive (e.g., baby category), neutral (e.g., clothing category) or negative (e.g., dirty things category), as determined by normative ratings. After a 48 h delay, all participants completed a category-cued recall task on their own; prior to this, a subset of participants completed a collaborative category-cued recall task with other participants from the same age group (young or older adults). See Methods for complete description of procedures.

This experiment took place across 2 days, separated by a 48 h delay for all participants. All parts of the experiment were completed online. The participants were first asked to provide their consent and to fill out demographic surveys. They were then redirected to a website (Pavlovia) to begin the study phase of the task, which had been programmed in PsychoPy. Participants saw a series of slides on the screen, and each slide contained a picture of an item, the verbal label for the item, and a category label at the top of the screen (see Figure 1 below). They rated the items on a scale from one to five of how well they thought the object fits into the category label; this task ensured they were paying attention to each slide. Each slide was shown for 3 sec, with a 1 sec fixation cross in between each slide. The entire study phase lasted approximately 35 min. All participants completed this phase on their own (individual study). This was an incidental encoding task, and participants were not aware that the second day of the experiment would include a memory test. At the end of the study phase, participants were provided a reminder to return online in 48 h to complete the second day of the experiment.

On the second day of the experiment, those in the collaborative condition joined a zoom call 48 h after the study phase. Participants were assigned to groups of adults of the same age group (all younger or all older adults). When the experimenter admitted a participant into the zoom room, they were assigned to a breakout room where they changed their Zoom name to an assigned letter ID (A, B, C or D); they were referred to by that initial by the other members of their retrieval group. After all participants returned to the main Zoom room, where they could see one another, they were told by the experimenter that they were going to work together with the other group members to take a memory test. Participants saw the label of three categories on the screen (e.g., actions, flowers, dirty things), and they were required to discuss with the others to try to recall as many of the items from those categories as they could. By presenting the labels of three categories at the same time, representing categories of different valences, we encouraged recall that was intermixed instead of blocked with reference to valence. If more than 2 min passed, they were encouraged by the experimenter to conclude the discussion of that set of categories. In this way, they went through all of the studied categories of information. The experimenter stayed on the zoom call and typed their responses for them on an excel sheet that all participants could see, using the screen-share function within Zoom. The instructions highlighted that the group did not have to reach consensus—if one participant thought a word was presented and others didn't agree, the experimenter would still type it in.

An individualretrieval phase immediately followed the collaborative session for those in the collaborative group, or was conducted as a stand-alone phase 48 h after the study phase for those in the individual condition. This individual retrieval phase was the main dependent measure of interest. Participants were directed to REDCap (via email for those in the individual condition or via the Zoom chat for those in the collaborative memory condition) to perform this memory retrieval test. The link took them to a page where category names were organized in the same triads as seen in the collaborative session (e.g., tools, toxic chemicals, toys), once again to encourage recall that was intermixed with reference to valence. They were then asked to write all the words that they remembered as presented 2 days earlier from the three categories in a text box below the categories. They could recall the words in any order, and there was no time limit given. The task was self-paced and after they recalled all the words from one triad, they could move on to the next triad. Participants could also go back to prior triads if needed.

## 3 Results

Data are available on OSF: https://osf.io/59nwr(Masterfile_SampleAnalyzed.csv). We analyzed recall performance from the final retrieval memory test for all participants using the 36 best categories (12 of each valence), using an ANOVA with valence as a within-subject factor, and age group (young, older) and collaborative condition (individual, collaborative) as between-subject factors. When effects were significant in the ANOVA, we then conducted follow-up t-tests with Tukey correction.

**Figure 2:**
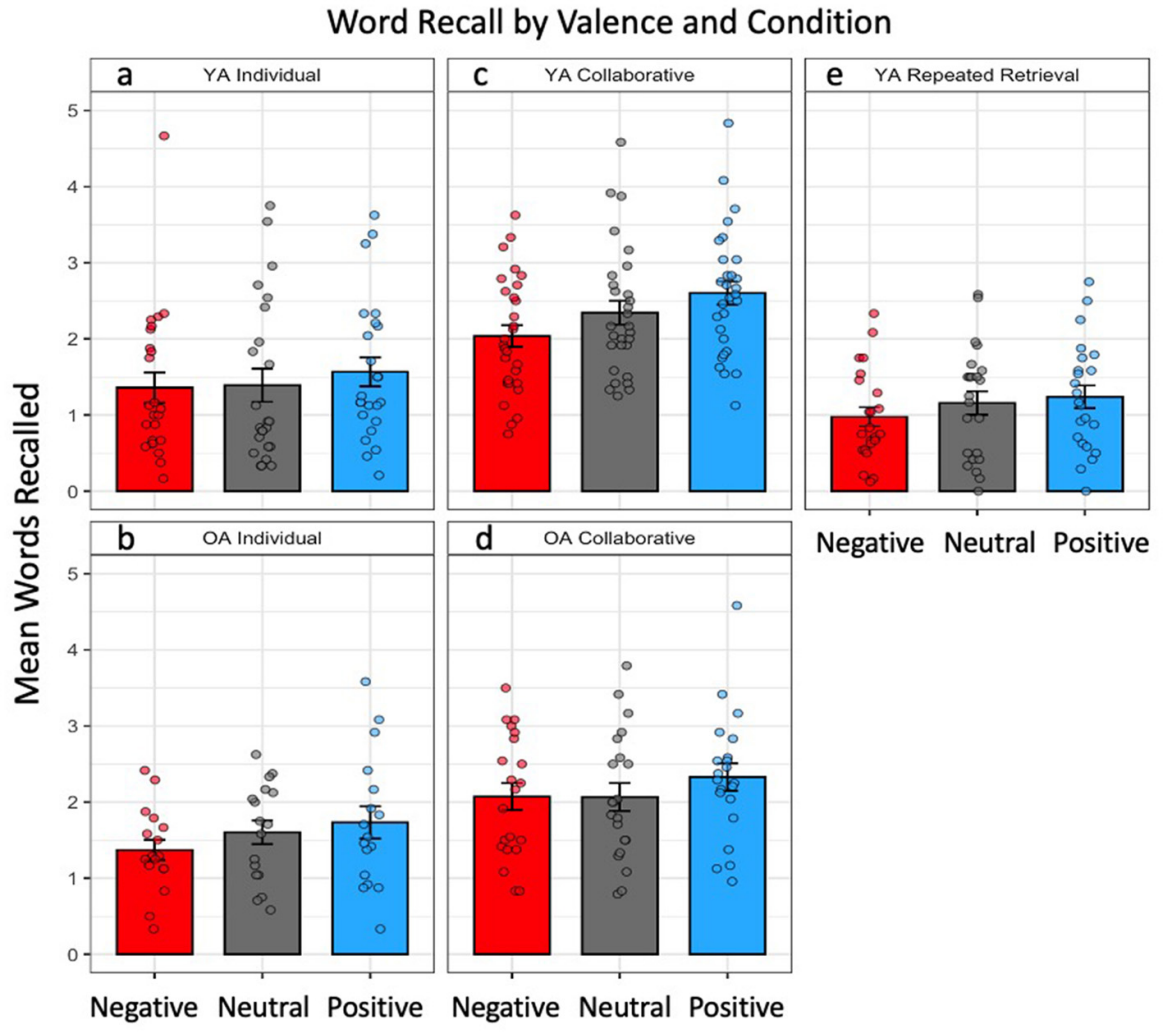
Mean words recalled per category on the final individual recall task, as a function of category valence, age group, and collaborative condition. Younger adults (YA) who had previously collaborated with other YA showed a positive memory bias **(C)**. This bias was significantly stronger than in YA who only recalled items individually (**A** and **E**). Older adults (OA) showed a positive memory bias regardless of whether they had collaborated with other OA previously; however, OA positive recall was better than neutral recall only after collaboration (**D**) and not with only individual recall (**B**).

The ANOVA revealed a main effect of condition [*F*(1,87)=18.4, *p* < 0.001, $\eta_{p}^{2}$ = 0.17], with better memory after collaboration than after individual recall, and a main effect of valence (*F*[2,174] = 24.1, *p* < 0.001, $\eta_{p}^{2}$ = 0.22), qualified by a significant valence X age group X condition interaction [*F*(2,174) = 4.0, *p* = 0.02, $\eta_{p}^{2}$ = 0.04]. There was no main effect of age group [*F*(1, 87) = 0.015, *p* = 0.90]. The pairwise comparisons revealed no significant differences between the three valences in the young adult individual condition, although there was marginally better memory for positive compared to negative (*t*[23] = 2.07, *p* = 0.05) or neutral (*t*[23] = 2.04, *p* = 0.053) content (see Figure 2a). In the collaborative condition, young adults showed a gradient effect of valence on memory (positive > neutral > negative): both positive (*t*[28] = 6.52, *p* < 0.001) and neutral (*t*[28] = 4.34, *p* < 0.001) items were significantly better recalled than negative items, and positive items were significantly better recalled than neutral (*t*[28] = 2.57, *p* = 0.016; see Figure 2c). Another way to consider this pattern is that while collaboration enhanced young adults' memory for all valences, it enhanced memory for positive valence to the greatest extent. Older adults in the individual condition recalled more positive (*t*[16] = 2.96, *p* = 0.009) and neutral (*t*[16] = 2.25, *p* = 0.038) items than negative items; there was not a significant difference between recall of positive and neutral items (*t*[16] = 0.95, *p* = 0.35; see Figure 2b). Older adults in the collaborative condition recalled positive items significantly better than negative (*t*[20] = 2.61, *p* = 0.017) and neutral items (*t*[20] = 2.96, *p* = 0.008); there was no significant difference between recall of negative and neutral items (*t*[20] = 0.07, *p* = 0.95; see Figure 2d).

We also tested a linear mixed-effects regression model as a confirmatory test to better partition subject and category variability, thereby ensuring that the observed effects in our standard ANOVA approach were not attributable to unmodeled subject-level variance or idiosyncratic differences between categories. To validate the initial ANOVA findings, we tested the mixed-effects model with all predictors entered simultaneously, maintaining a consistent model structure that was predefined by our experimental design. The model included fixed effects for valence, age group, and collaboration condition (fully crossed), as well as random intercepts for subject and category. The dependent variable was the total sum score of recall for each category. This analysis confirmed a main effect of collaboration condition [*F*(1,87) = 18.48, *p* < 0.001], but the main effect of valence was no longer significant now that the model accounted for variability across categories [*F*(2,33.07) = 0.711, *p* = 0.499], and there was still no main effect of age group (*F*[1,87] = 0.015, *p* < 0.904). Crucially, the three-way interaction of valence *x* age group *x* collaboration condition remained significant (*F*[2,3144] = 4.00, *p* = 0.018), with follow-up tests showing a significant two-way interaction of valence by collaboration among younger adults [*F*(1,3144) = 4.20, *p* = 0.015] but not among older adults [*F*(1,3144) = 1.22, *p* = 0.297]. Thus, collaboration enhanced younger adults' memory positivity but not older adults' memory positivity (see Figure 2).

### 3.1 Controlling for additional retrieval

A difference between the Individual and Collaborative condition was that those in the Collaborative condition had a second opportunity to retrieve content. To clarify whether this second retrieval, rather than collaboration, might have led to the increased positivity bias in younger adults, we enrolled an additional 23 younger adults (11 F, 12 M) between the ages of 18 and 35 (mean age = 28.7 years, SD = 5.0 years) in a “Repeated Retrieval” condition. These participants performed the same incidental encoding task and individual-retrieval task as the other groups, with the same 48-h delay, but just prior to the individual-retrieval task they performed an additional category-cued recall task which was modeled after the retrieval in the collaborative group condition but was completed alone within an online study (i.e., without a zoom call). Results from this condition are presented in Figure 2e.

A repeated-measures ANOVA comparing young adults' recall in the Individual Recall condition with the Repeated Retrieval condition revealed only a main effect of valence [*F*(2,90) = 6.057, *p* = 0.003, $\eta_{p}^{2}$ = 0.119] with no main effect of condition [*F*(1,45) = 1.75, *p* = 0.19, $\eta_{p}^{2}$ = 0.038] and no interaction between valence and condition [*F*(2,90) = 0.627, *p* = 0.53, $\eta_{p}^{2}$ = 0.014]. *Post-hoc* comparisons confirmed that there was no significant difference in recall for neutral, *t*(45) = 0.868, *p* = 0.95, negative, *t*(45) = 1.604, *p* = 0.60, or positive, *t*(45) = 1.355, *p* = 0.75 content between the Individual and the Repeated Retrieval. Thus, there was not an increase in the positivity bias just as a function of having repeated retrieval.

By contrast, in a repeated-measures ANOVA comparing young adults' recall in the Repeated Retrieval and Collaborative conditions, there was not only a main effect of valence, (*F*[2,100]=21.79, *p* < 0.001, $\eta_{p}^{2}$ = 0.304) but also a main effect of condition (*F*[1,50] = 36.5, *p* < 0.001, $\eta_{p}^{2}$ = 0.422), and an interaction between valence and condition (*F*[2,100] = 2.95, *p* = 0.05, $\eta_{p}^{2}$ = 0.055). As indicated by the main effect of condition, *post-hoc* comparisons confirmed that collaboration led to better recall of all valences compared to repeated retrieval alone; however, as indicated by the interaction between valence and condition, the strength of the memory benefit from collaboration (i.e., the magnitude of the *t* statistic) was greatest for the positive content, *t*(50)=6.275, *p* < 0.001, compared to the negative content, *t*(73) = 5.43, *p* < 0.001, or the neutral content, *t*(73) = 5.32, *p* < 0.001.

These patterns were replicated when using a linear mixed-effects regression model with fixed effects for valence, age group, and collaboration condition (fully crossed), as well as random intercepts for subject and category. In the analysis comparing Individual and Repeated Retrieval, this model revealed no significant effect of Condition, *F*(1, 46.90) = 1.82, *p* = 0.18, and no Condition × Valence interaction, *F*(2, 1609.94) = 0.78, *p* = 0.46; whereas in the model comparing Collaborative and Repeated Retrieval, this model revealed a significant main effect of Condition, *F*(1, 51.73) = 37.56, *p* < 0.001, and a significant Condition x Valence interaction, *F*(2, 1784.93) = 2.99, *p* = 0.05.

Together, these results support the conclusion that it is collaboration, rather than repeated retrieval alone, that led to a change in the valence of content recalled by younger adults.

## 4 Discussion

The goal of this study was two-fold. First, we sought to better understand conflicting findings in the literature with regard to whether collaboration among younger adults enhances memory negativity (Choi et al., 2017) or leads to more positive memories (Maswood et al., 2019). Second, we explored how collaboration affects older adults' emotional memory patterns and whether collaboration affects older adults' valence biases in memory. The results of this experiment suggest that, when memories are cued in a way that encourages mixing of differently valenced content, collaboration enhances positive memory biases, especially for younger adults. We found no significant effect of collaboration on valence biases in memory for older adults, although this may be because their memories already show a positive memory bias after individual retrieval. In this discussion, we elaborate on these results and their potential implications, while also emphasizing the limitations of this preliminary investigation into the effects of collaboration on young and older adults' collaborative memories.

### 4.1 Positivity effects with aging and after collaboration

Younger adults who always recalled content on their own were the only group to show no significant valence memory bias. Older adults who recalled content on their own showed a positive memory bias, consistent with prior work (Mather and Carstensen, 2005) and younger adults who had previously collaborated also showed a positive memory bias. Thus, both aging and collaboration can separately lead to positive memory biases.

Future work will be needed to clarify whether there is an additive or interactive effect of aging and collaboration on positive memory biases. Although the ANOVA results revealed no significant differences between older adults in the individual and collaborative condition, the *post-hoc t*-tests revealed a potentially interesting difference. When older adults had always recalled individually, their positive and neutral recall did not differ, and both were higher than their negative recall; it was only after collaboration that their positive recall was greater than their neutral recall. This may suggest that collaboration increases the positivity of memories even for older adults, who already show a tendency toward more positively biased memories.

The same encoding task used here has previously revealed negative memory biases after collaboration (Choi et al., 2017; Kensinger et al., 2016). The critical difference we implemented here was to cue multiple categories together, cuing categories of different valences at the same time. In other words, the category triad would often require categories of different valences to be recalled simultaneously. In prior research using the current encoding task, the retrieval phase did not encourage this type of intermixed recall of differently-valenced content; participants either performed a free recall task that may have led to a default clustering by semantic category or by valence, or a recognition memory task in which they saw single items and judged whether each was old or new (Kensinger et al., 2016; Choi et al., 2013, 2017). The divergence in results across studies suggests that a positive memory bias after collaboration may arise when tasks encourage the intermixed retrieval of different valences of information. Interestingly, even when younger adults recalled individually, they did not show a typical negativity bias, and numerically showed evidence of a positive memory bias; this may suggest this intermixing of valences affects the memories of those retrieving individually, though the effect is exaggerated after collaborative recall, leading to a significant positivity bias. This finding is consistent with why collaborative recall of autobiographical memories encourages positive bias, because the event or the story structure guides recall of units that are likely to be intermixed in valence to serve the event narration.

Why might positivity effects arise under these circumstances? One answer may relate to the different effects that collaboration can have on memory. It has been demonstrated that collaboration can affect both the content and the structural organization of memory (Congleton and Rajaram, 2014; Greeley and Rajaram, 2023). It may be easier to reveal structural organization impacts when content from multiple different valence categories can be retrieved at once. Thus, this type of retrieval design may be optimal for revealing ways that collaboration pulls apart positive from negative and neutral valence in memory organization. Another answer may relate to the social consequences of collaboration that led to the positivity bias in both age groups. Maswood et al. (2019) noted that their results—that young adults who collaborated when recalling emotional autobiographical memories later remembered these events with a more positive emotional tone compared to individuals who recalled alone—were generally consistent with those of Pasupathi (2003) and Skowronski et al. (2004) who reported that emotions faded for participants recalling negative memories after social rehearsal. Thus, Maswood et al. (2019) suggested that collaboration may serve as a form of emotion regulation. Social psychology research suggests that social interactions and collaboration can positively influence mood and learning outcomes. Group interactions have been shown to sustain positive moods and diminish negative ones, highlighting the affective benefits of collaboration (Park and Hinsz, 2015). The current results may provide further evidence of this emotional regulatory function of collaboration, and may further suggest that online interactions are sufficient to elicit this function of collaboration.

### 4.2 Limitations of this research

A main limitation of the present study is that it was powered to detect only medium-to-large effect sizes. This sample size was adequate to allow us to examine *which direction* of valenced memory bias results when younger adults collaborate and to explore whether there was a *large age difference* in the effect of collaboration on valenced memory bias. However, our sample size was insufficient to detect small-to-medium effects of collaboration on older adults' memories or interactions among the tested variables. As noted earlier, there is more work to be done to clarify whether age and collaboration have additive or interactive effects on positive memory biases.

Another limitation of the present study is that the collaboration all took place online. The nature of social interactions online can differ from those that take place in person. This is not in itself a limitation, as many modern-day conversations take place online (Rajaram, 2024b), but it is important to note that the results may not generalize to in-person collaboration. The online nature of the task also changed the set of participants who could take part in the study: It broadened the sample in some ways (e.g., including those who could not travel to Boston College for in person testing), but it restricted the sample in other ways (e.g., including only those older adults with access to, and comfort with, technology). Another design limitation is that, to avoid discarding data, we allowed groups of two, three, or four to proceed. Although the results were unchanged when we removed the two dyads from analyses, and when multi-level modeling was used to include group as a factor, it is still possible that there are subtle effects of group size that modulate the reported patterns.

In addition, the sample of participants recruited was not representative of the broader population; as noted in Table 1, participants were highly educated, and the younger adult sample had a disproportionate number of females. Having this more homogenous sample along some dimensions may affect the social consequences of collaboration. Many of the recruited participants also would have had high familiarity with memory tasks, either because they are younger adults who are, or recently were, students, or because they are older adults who engage in many psychological science studies. This familiarity may have affected the way they approach the task.

Finally, although we propose that the way we tested recall may have led to the revelation of the positive memory biases after younger adults' collaboration, we did not include an alternate way of testing memory. It is therefore possible that positive memory biases would have been revealed with online collaboration even if memory had been tested using the methods of (Choi et al., 2013, 2017). Future work will be needed to clarify the conditions under which positivity biases are revealed after young adults collaborate together. Nevertheless, the present results are important in revealing that positivity biases can result from collaboration not only when it is autobiographical experiences that are being discussed (Maswood et al., 2019) but also when it is more impoverished laboratory-presented stimuli being recalled. A key to linking these divergent patterns may be retrieval of information that is intermixed in valence, as arranged in the present study. Thus, despite the limitations of this research, this investigation is important for providing preliminary evidence that both aging and also collaboration can shift the valence of information recalled toward the recall of positive information.

## Data Availability

The datasets presented in this study can be found in online repositories. The names of the repository/repositories and accession number(s) can be found below: https://osf.io/59nwr/.
