# Supplementary material for: Preliminary evidence that remembering together can help younger adults recall the positive
Source: Front Cognit. 2026 Jun 22;5:1814611. doi: 10.3389/fcogn.2026.1814611 (PMC13333520; doi:10.3389/fcogn.2026.1814611)

**Supplementary Materials**

***Analyses with all 15 categories***

Although the main analyses focused on analyses with the 12 categories of each valence that best fit the valence and arousal criteria, we also conducted analyses with all 15 categories of each valence.

This ANOVA revealed a main effect of valence (F(2,174)= 7.0, p<.001, η_p_^2^ =0.07) and a main effect of condition (F(1,87)=20.5, p<.001 η_p_^2^ =0.19). There was no significant valence X age group X condition interaction (F(2,174) =1.6, p=0.21, η_p_^2^ =0.018). There was no main effect of age group (F(1, 87)=0.004, p=.95) nor any two-way interactions (all F < 2.02, all p > .13, all η_p_^2^ < .025).

Despite the lack of significant interactions in the ANOVA, the pairwise comparisons revealed a pattern broadly similar to the one described in the body of the manuscript, with a positive memory bias existing for young adults only after collaboration. There were no significant differences in memory across the valence conditions when young adults recalled individually (all t(23)<1, all p>0.30); however, when younger adults collaborated, positive content was remembered better than neutral content (t(28)=4.23,p<.001) and neutral content was remembered better than negative content (t(28)=3.78,p<.001). For older adults, there were no significant differences in memory across valences when they recalled individually (all t(23)<1.4, all p > 0.15). When they collaborated, there was a marginal tendency for positive content to be recalled better than neutral content (t(28) = 1.72, p = .102), with other valence differences nonsignificant (t(28) < 1.3, p > .22).

Figures showing the pattern of results for all 15 categories are shown below. Note that these figures show the summed recall across all categories of a certain valence, whereas the figure in the main body of the text shows the average recall across categories of a certain valence, which is why the y-axis on the figures differ in scale.


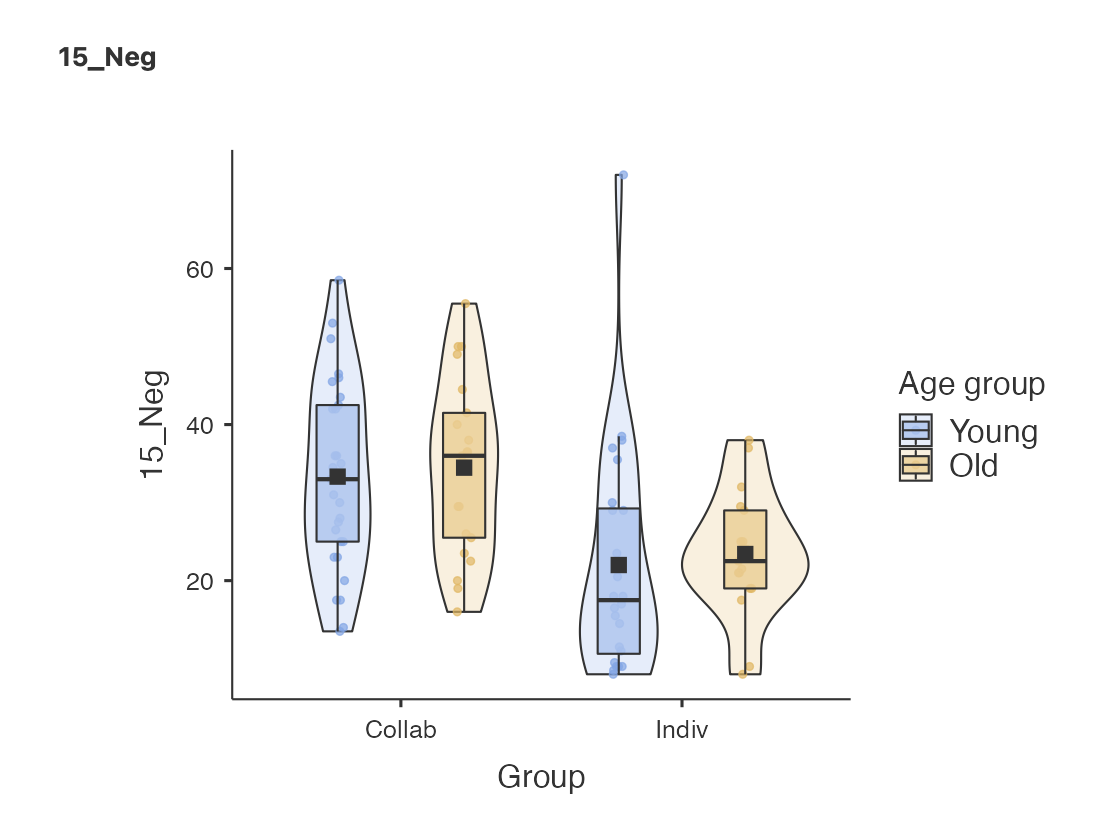


**
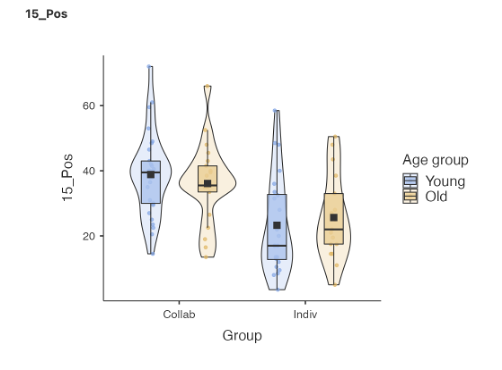
**
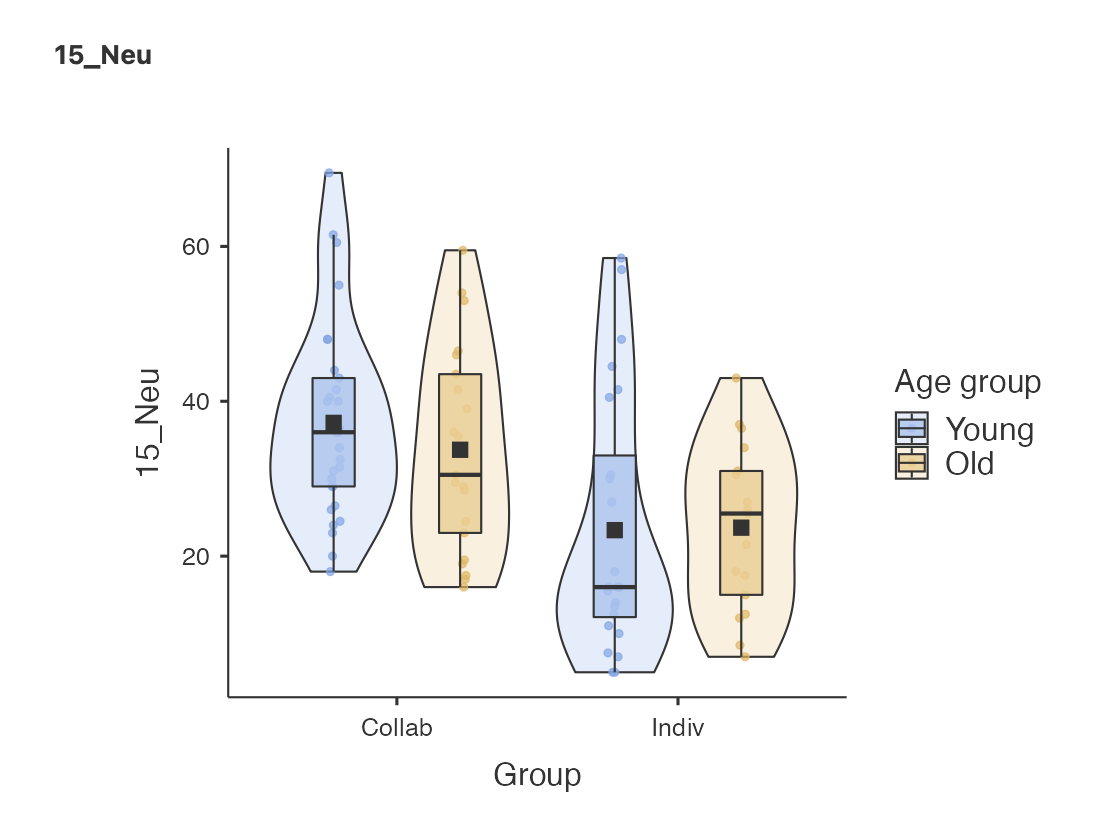

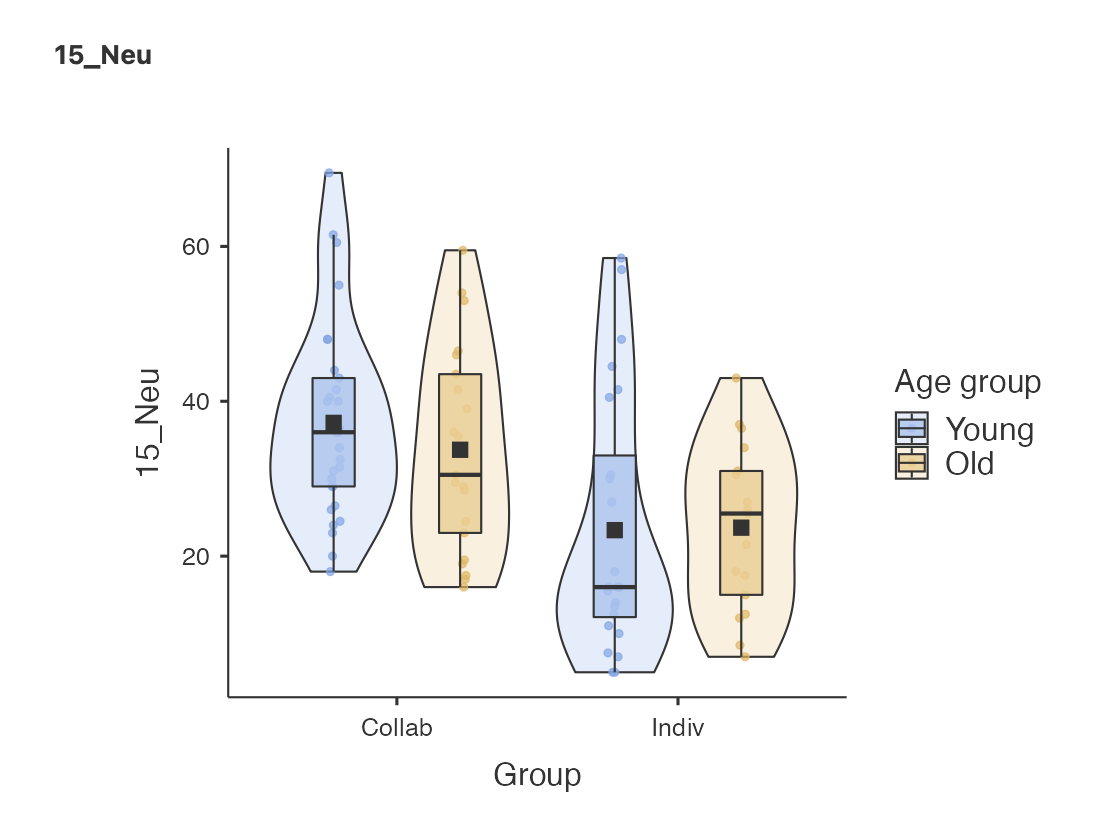

Supplement: Supplementary file 1 [file Supplementary_file_1.docx]
